# A novel multiplexed blood biomarker approach for improved stratification of dementia

**DOI:** 10.1002/alz70856_106343

**Published:** 2026-01-08

**Authors:** Souvik Modi, Shareefa Thekkan, Zhimeng Zhang, Dhivya Venkat, Andreas Jeromin

**Affiliations:** ^1^ Esya Labs, London, United Kingdom; ^2^ ESYA labs, London, London, United Kingdom; ^3^ Esya labs, London, United Kingdom; ^4^ Esya labs, Dallas, TX, USA

## Abstract

**Background:**

Blood‐based biomarkers have been commonly employed to identify certain neurodegenerative disorders. While these markers independently provide insights into neuropathology, the potential of a single multiplex panel to accurately diagnose and differentiate types of dementia remains unexplored. Utilizing a novel multiplex proteomic method, we screened a set of biomarkers specific to Alzheimer's disease (AD), frontotemporal dementia (FTD), and vascular dementia (VAD). These biomarkers were then integrated into a multi‐class machine learning model for classification. Our approach highlights the feasibility of a multiplex blood‐based biomarker panel to distinguish between different types of dementia, paving the way for more accurate and early diagnosis.

**Method:**

We screened dementia‐specific biomarkers from 120 diverse CNS‐related proteins using the Nucleic Acid Linked Immuno‐Sandwich Assay (NULISA) on samples from 150 participants (18 controls, 92 AD, 20 FTD, and 20 VAD). Posterior probabilities were calculated using Linear Discriminant Analysis, and diagnostic performance was compared using the Area Under the Curve (AUC).

**Result:**

We identified that pTau217 and GFAP levels were significantly different between control and AD groups. Similarly, SFTPD and HTT levels showed significant differences between control and VAD groups, while GFAP, SFRP1, and phosphorylated TDP43 levels were significantly different between control and FTD groups. When comparing FTD and AD groups, we found that FGF2, phosphorylated TDP43, SMOC1, phosphorylated SNCA, and HTT were differentially abundant, whereas pTau217 and acetylcholinesterase were significantly different between AD and VAD groups. Additionally, PGK1 and NRGN levels were significantly different between FTD and VAD groups. The AUC of a multi‐analyte assay involving seven key biomarkers was 0.908 for distinguishing control from all dementia types, 0.918 for control versus AD, 0.872 for control versus FTD, and 0.897 for control versus VAD. For dementia subtype differentiation, the AUC was 0.791 for AD versus FTD, 0.719 for AD versus VAD, and 0.800 for FTD versus VAD.

**Conclusion:**

Our multi‐analyte approach identified key markers that are differentially abundant across various forms of dementia. A combination of seven distinct plasma biomarkers demonstrates potential for a comprehensive evaluation panel for dementia diagnosis. This proof‐of‐principle platform could significantly contribute to the further development of dementia subtyping, monitoring disease progression, and complementing drug development efforts.

